# Methodology for adapting a co-created early childhood development intervention and implementation strategies for use by frontline workers in India and Guatemala: a systematic application of the FRAME-IS framework

**DOI:** 10.1080/16549716.2024.2338324

**Published:** 2024-05-10

**Authors:** Amruta Bandal, Sara Hernández, Revan Mustafa, Karyn Choy, Namrata Edwards, Magdalena Guarchaj, Marinés Mejía Alvarez, Anushree Sane, Scott Tschida, Chetna Maliye, Ann Miller, Abhishek Raut, Roopa Srinivasan, Morgan Turner, Bradley H. Wagenaar, Ilgi Ertem, Maria del Pilar Grazioso, Subodh S. Gupta, Vibha Krishnamurthy, Peter Rohloff

**Affiliations:** a Department of Community Medicine, Mahatma Gandhi Institute of Medical Sciences, Sewagram, India; b Center for Indigenous Health Research, Maya Health Alliance, Tecpán, Guatemala; c Department of Pediatrics, Acıbadem Maslak Hospital, Istanbul, Turkey; d Early Childhood Development and Disabilities, Ummeed Child Development Centre, Mumbai, India; e Proyecto Aigle Guatemala, Guatemala City, Guatemala; f Department of Global Health and Social Medicine, Harvard Medical School, Boston, USA; g Department of Global Health, University of Washington, Seattle, USA; h Department of Epidemiology, University of Washington, Seattle, USA; i Developmental-Behavioral Pediatrics Division, Department of Pediatrics, Faculty of Medicine, Ankara University, Ankara, Turkey; j Division of Global Health Equity, Brigham and Women’s Hospital, Boston, USA

**Keywords:** Early child development, participatory research, implementation science, LMIC, rural health care, Anganwadi worker, community health worker

## Abstract

There is little evidence on optimizing the effectiveness and implementation of evidence-based early childhood development (ECD) interventions when task-shifted to frontline workers. In this Methods Forum paper, we describe our adaptation of the International Guide for Monitoring Child Development (GMCD) for task-shifting to frontline workers in Guatemala and India. In 2021–2022, implementers, trainers, frontline workers, caregivers, and international GMCD experts collaborated to adapt the GMCD for a task shifted implementation by frontline workers. We used an eight-step co-creating process: assembling a multidisciplinary team, training on the existing package, working groups to begin modifications, revision of draft modifications, tailoring of visual materials and language, train-the-trainers activities, pilot frontline worker trainings, final review and feedback. Preliminary effectiveness of adaptations was evaluated through narrative notes and group-based qualitative feedback following pilot trainings with 16 frontline workers in India and 6 in Guatemala. Final adaptations included: refining training techniques to match skill levels and learning styles of frontline workers; tailoring all visual materials to local languages and contexts; design of job aids for providing developmental support messages; modification of referral and triage processes for children in need of enhanced support and speciality referral; and creation of post-training support procedures. Feedback from pilot trainings included: (1) group consensus that training improved ECD skills and knowledge across multiple domains; and (2) feedback on ongoing needed adjustments to pacing, use of video-based vs. role-playing materials, and time allocated to small group work. We use the Framework for Reporting Adaptations and Modifications to Evidence-based Implementation Strategies (FRAME-IS) framework to document our adaptations. The co-creating approach we use, as well as systematic documentation of adaptation decisions will be of use to other community-based early childhood interventions and implementation strategies.

## Paper Context

- **Main findings:** The International Guide for Monitoring Child Development, an early childhood development support and monitoring tool, was successfully adapted for use by frontline workers in rural India and Guatemala.
- **Added knowledge:** Our Methods Forum paper uses a detailed framework to document the collaborative, co-creating process used and the adaptive decisions taken.
- **Global health impact for policy and action:** Evidence on how best to adapt and optimize early childhood interventions for frontline workers will be useful or scaling up support for children globally.

## Background

The Sustainable Development Goals (SDG) advocate globally for a holistic focus on child health, nutrition, early childhood development (ECD), and access to education [1]. To support the Sustainable Development Goals, the World Health Organization and UNICEF have developed the Nurturing Care Framework, which encourages countries to implement synergistic interventions to support child wellness. To be effective and scalable, such interventions must be community-based, family-centered, culturally adaptable, and responsive to the needs of children with development difficulties [2,3].

One such comprehensive intervention package is the International Guide for Monitoring Child Development (GMCD) [4]. However, this package has up to now primarily been used by healthcare professionals with high levels of literacy and education. Limitations to the availability of such professionals in Low and Middle Income Countries (LMICs) has prompted efforts to task-shift the GMCD intervention to frontline workers. Our group has recently adapted the GMCD package for task-shifting to rural frontline workers, and we are conducting a cluster-randomized effectiveness trial of this adapted intervention in India and Guatemala [5].

Task-shifting comes with significant challenges, including the need to carefully adapt training strategies and interventions to the needs and competencies of frontline workers. There is significant literature on task-shifting to frontline workers, including for HIV care, maternal-child health, mental health, and chronic disease management [6–10]. However, very little of this literature examines the concrete steps required to adapt such interventions.

In this Methods Forum paper, we describe the process of ‘*co-creating adaptation*’ that we used to conduct a task-shifting adaptation of the GMCD intervention for use by frontline workers in India and Guatemala. Co-creating approaches are essential to advancing SDG goals, because they support user engagement, cultural sensitivity and local ownership while paying attention to fidelity and reliability [11]. We document our process with the Framework for Reporting Adaptations and Modifications to Evidence-based Implementation Strategies (FRAME-IS) tool [12].

## Context and study setting

The GMCD is a comprehensive early intervention package designed for use with both typically developing children and children with developmental difficulties [13–15]. The GMCD method is based on an interview with the primary caregiver which uses open-ended questions and active listening to establish rapport. In a typical GMCD visit, the provider and caregiver work together to explore caregiver’s concerns around their child’s development, monitor the child’s developmental functioning, explore strengths and risk factors, engage in problem-solving to address risk factors, provide individualized recommendations for developmental support and identify referral needs.

The GMCD has been translated into more than 15 languages and service providers in more than 30 countries have been trained in its use [16]. A key strategic effort of all prior GMCD country implementations has been the training of a local cadre of GMCD trainers who would then lead training and certification of local healthcare providers (physicians and other professionals) as part of scaling-up activities. The base training package for these providers has been a two-day intensive interactive training, with an additional four-day version for frontline workers in India who have already received formative training on the WHO/UNICEF Care for Child Development intervention [13,15,17].

We recently began a cluster-randomized effectiveness trial of the GMCD intervention delivered by frontline workers to caregivers of children aged 0–24 months at enrollment. This study protocol has been published previously. The protocol was designed to explore the differential effectiveness of task-shifting the GMCD to frontline workers in two very different contexts (public vs private sector) [5]. In Guatemala the primary implementing partner is Maya Health Alliance, a nongovernmental organization working with Indigenous Maya populations, and each cluster is a community-based child health program run by a nongovernmental frontline worker. In India, the primary partners are the Mahatma Gandhi Institute of Medical Sciences (MGIMS) and Anganwadi workers from the government’s Integrated Child Development Services (ICDS) program. Each cluster in India is comprised of 2–3 Anganwadi child centers run by its governmental frontline workers.

While preparing for our clinical trial and pilot testing the existing GMCD training packages for this project with our cohorts of frontline workers with no prior ECD experience in Guatemala and India, we discovered many challenges including those related to educational and reading levels and lack of familiarity with classroom-based didactic learning. It was clear that a full-scale adaptation would be needed. This effort was conducted from June 2021 to December 2022 and described in the following sections.

## Co-creating adaptation model for frontline workers

**Figure 1.**
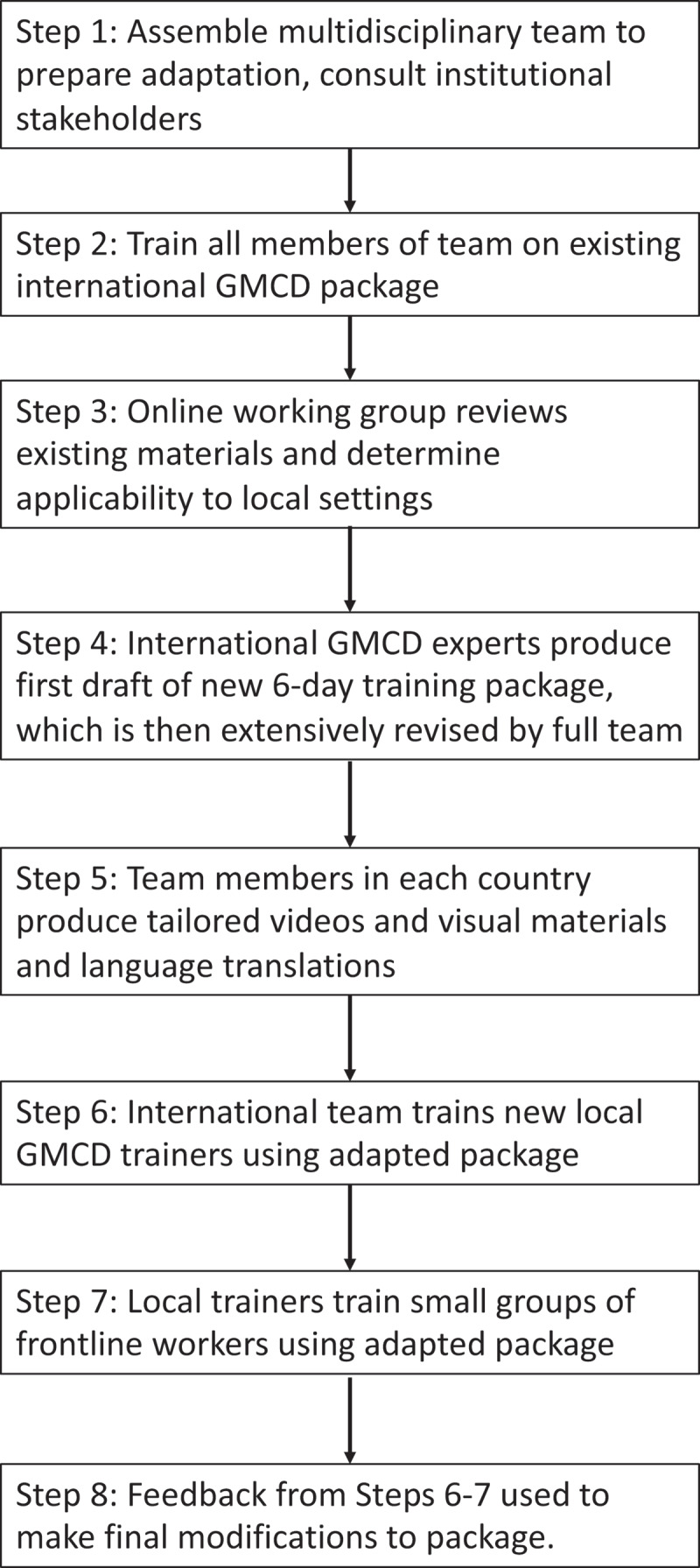
Eight steps to co-create and iteratively test adaptations to the GMCD for use by frontline workers.

The steps that we followed for the co-creating adaptation are summarized here and in Figure 1. Our multidisciplinary team included (1) researchers from our team; (2) trainers of frontline workers; (3) frontline workers; (4) caregivers; and (5) international GMCD experts. Common working knowledge was developed by training all members of the adaptation team on existing materials. Next, working together online, we conducted a first review of these materials to determine their applicability to local settings.

Upon this review, the team jointly decided to expand existing two- and four-day training to six-days for frontline workers. The international experts provided a first draft of the new six-day package which was then extensively revised based on input from both country sites. After revision, team members in each country site produced tailored video and photo materials, as well as language translations of materials. Next, international experts provided online training of trainers to local teams using the new materials. These local trainers at each site then conducted their own trainings of small groups of frontline workers to pilot the new materials, and feedback from the frontline workers as well as local trainers was used to make final adjustments. These training sessions were observed by international GMCD experts, providing another layer of feedback. After the pilot training, final inputs and feedback from frontline workers, trainers and international GMCD experts were synthesized and incorporated into the training package.

Throughout the adaptation process, key decisions were documented using meeting and working-group notes. These decisions were thematically grouped using the FRAME-IS Framework. A summary of these decisions is given in Table 1. The most salient adaptation considerations and decisions are highlighted in more detail in the following text.

**Table 1.** Summary of adaptation decisions and context for implementing the GMCD with frontline workers using FRAME-IS^1^.

FRAME-IS module	Summary
***Module 1: Brief description of evidence-based practice, implementation strategy, and modification***	
Practice being implemented:	-The international Guide for Monitoring Child Development (GMCD) Early Intervention Package
**Implementation strategy being modified:**	-Task-shifting intervention to frontline workers - Intervention moved from primarily clinic-based to home-based setting.
**Modifications being made:**	-Tailoring of training content and developmental support materials to be used during home visits for local populations -Expansion of training length and content for frontline worker implementers -Development of frontline worker-appropriate job aids -Strengthening post-training support strategies
Reasons for modification:	-To task-shift the GMCD implementation to frontline workers with relatively low literacy and educational attainment for use in rural communities in India and Guatemala.
*Module 2: What is modified?*	
Content	-Tailoring (local languages, photos, videos) -Flipchart for delivering developmental support recommendations (replacing manuals and digital app) -Local algorithms for referral guidelines and home visit frequency -Addition of local resources providing early intervention services to the referral algorithm -Development of frontline worker-appropriate job aids - Added practice sessions with caregivers supervised by trainers
Training	-Expansion of training from 2–4 to 6 days -Post-training support strategies
Context	-Developmental monitoring and support to be largely done in home visits rather than clinic settings
Personnel	-Delivery of GMCD intervention by frontline workers with limited prior ECD experience
***Module 3a: What is the nature of the content, evaluation, training modification?***	
**Tailoring**	-Local videos and photos from rural Maharashtra, India; and Chimaltenango, Guatemala showing the development of children and how caregivers support development -Language adaptations to Marathi (India) and Kaqchikel, K’iche’ and Tz’utujil (Guatemala)
**Changes in packaging/materials**	-Reformatting of developmental support recommendations to an easily-used flipchart format -Adding summary handouts of key ECD concepts for trainees -Reformatting of child and family triage and referral guidelines algorithm to a detailed table with triage groups based on consideration of risk factors - Algorithms for referral placement and visit frequency designed as per local available resources -Training augmented with concepts related to brain development, developmental milestones, caregiver mental health, calculation of chronological age in months and corrected age for prematurely born infants. -Development of frontline worker-appropriate job aids (counseling flipchart for ensuring consistency in delivery of developmental support recommendations, tools for calculation of chronological age and corrected age for prematurity)-
**Adding training elements**	-Added visual material to replace text-heavy training elements -Added interactive exercises and games related to theoretical content -Added interactive exercises and games related to technique and skills essential for GMCD users
**Condensing training pacing/timing**	-Condensed training time dedicated to exploring the science of brain development and published GMCD evidence -Condensed time dedicated to review of homework assignments
**Removing/skipping training elements**	-Removal of text-heavy training slides
**Lengthening training pacing/timing**	-Increased training time spent on learning to administer, code, and interpret GMCD developmental monitoring tool -Increased training time spent on learning developmental milestones -Augmented post-training support strategy, shifting from case-based discussion to more generalized longer-term support strategies
**Substitutions in training**	-Substituted a wellness-oriented caregiver mental health module for original module focusing narrowly on caregiver depression
**Breaking up/spreading out training content**	-Breaking up all sessions with short fun games to maintain focus and energy -Separating related training objectives into separate training sessions to improve comprehension (e.g. ‘coding on GMCD’ separated from ‘interpretation of GMCD results’)
**Loosening structure**	-Individualized supplementary training sessions for frontline workers with absences or time constraints
*Module 3b: Relationship to fidelity/core elements?*	Core elements and functions preserved
*Module 4a: What is the goal?*	-Increase reach and adoption of the GMCD by utilizing frontline worker cohorts in diverse rural communities in different countries -Decrease costs of GMCD implementation through task-shifting to frontline workers -Increase acceptability of the GMCD intervention through contextual adaptations -Improve fidelity of the GMCD intervention when implemented by frontline workers -Improve sustainability and decrease disparities by decentralizing implementation of GMCD and expanding access to rural communities
*Module 4b: What is the level of rationale for modifications?*	
Organizational	-Address lack of reach, adoption, and sustainability of ECD interventions in rural LMICs
Implementers and patients	-Address contextual differences and tailoring of GMCD training package
*Module 5a: When is the modification initiated?*	Planning phase: prior to start of randomized clinical trial
*Module 5b: Is the modification planned?*	Planned, proactive adaptation
*Module 6: Who participates in the decision to modify?*	
Program leaders and administrators	-Members of international GMCD development and training team; ICDS district leadership, India; leadership at Maya Health Alliance, Guatemala
Researcher	-International GMCD clinical trial investigator team
Clinicians and public health professionals	-GMCD trainers and clinicians at Ummeed Child Development Center (India); Mahatma Gandhi Institute of Medical Sciences (India); Maya Health Alliance (Guatemala)
*Module 7: How widespread is the modification?*	-Clinical trial implementation: ● 54 Anganwadi Centers (54 Anganwadi workers) in Wardha district, Maharashtra, India ● 12 Nongovernmental organizations (37 community health workers) in provinces of Sololá, Quiche, Sacatepéquez, Chimaltenango, Guatemala, and Suchitepéquez -Post clinical trial: ● Advocacy and networking efforts ongoing for regional scale-up in both countries

^**1**^**Bolded** FRAME-IS elements are discussed in more detail in the following narrative text.

### Module 1: brief description of evidence-based practice, implementation strategy, and modification

As outlined in Table 1 and Figure 1, our focus was to modify the GMCD implementation strategy for task-shifting to frontline workers and for a home visit-based model. The most important initial adaptation was to expand the length of GMCD intervention training to six days from prior two- and four-day versions. One day a week for six weeks was considered the maximum length of time away from work that could be feasible during normal operations by ICDS and nongovernmental organization leadership. A six-day training allowed for additional content on early brain development, developmental milestones, caregiver mental health and other risk factors as well as more interactive sessions. Additional supervised practice sessions with caregivers were added to each training day to build confidence and skill development. Several additional thematic areas from the adaptation are described in the following sections.

### Module 3a: tailoring

The clinical trial involves delivery of the GMCD intervention during home visits to families in rural areas of Maharashtra, India (Marathi speaking) and central Guatemala (Indigenous Maya, speaking Spanish as well as Kaqchikel, K’iche’ or Tz’utujil Mayan languages). All core GMCD components for use with families were translated by local teams into these languages using best practices, including back-translation [18].

**Figure 2.**
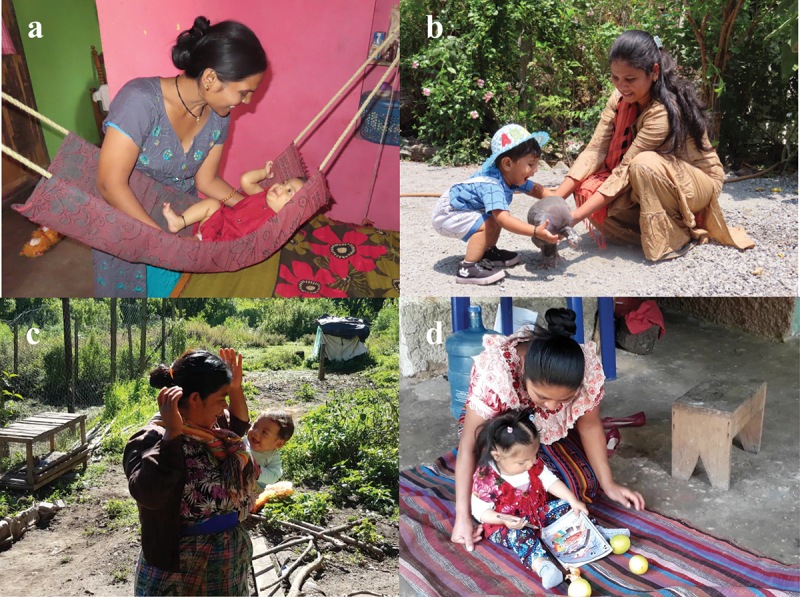
Example photos adapted for use by rural frontline worker cohorts for the GMCD early childhood development intervention in India (a, b) and Guatemala (c, d). (a) Mother talking with infant in a homemade swing (b) Mother encouraging play with a domestic animal (c) Mother engaging carried infant in peek-a-boo game (d) Mother playing with child on prepared play surface on outside patio.

For the purposes of adaptation, more important than just translation of text was ensuring that all caregiver messages and visual aids were also appropriately tailored to reflect the local environment, cultural worldviews, and context. Examples included photos and videos illustrating local home-based caregiving settings, play materials, and other important elements in the rural environment, like being in nature and interacting with domestic animals (Figure 2). The main strategy for arriving at these final contextual decisions was ensuring a multidisciplinary adaptation team including researchers and GMCD users from the intervention communities themselves, as well as multiple rounds of piloting and feedback with caregivers (Figure 1 and accompanying text).

### Module 3a: changes in packaging/materials (Job aids for frontline workers)

The GMCD early intervention package guides healthcare workers to provide individualized developmental support to caregivers based on the individual child’s current level of functioning rather than age-based recommendations. This individualized support had been embedded in a GMCD digital app developed prior to this study. However, the adaptation team decided not to use the app due to pilot work showing discomfort with and limited prior experience of frontline workers with app technology, as well logistical constraints on device acquisition and maintenance for the implementing partners.

**Figure 3.**
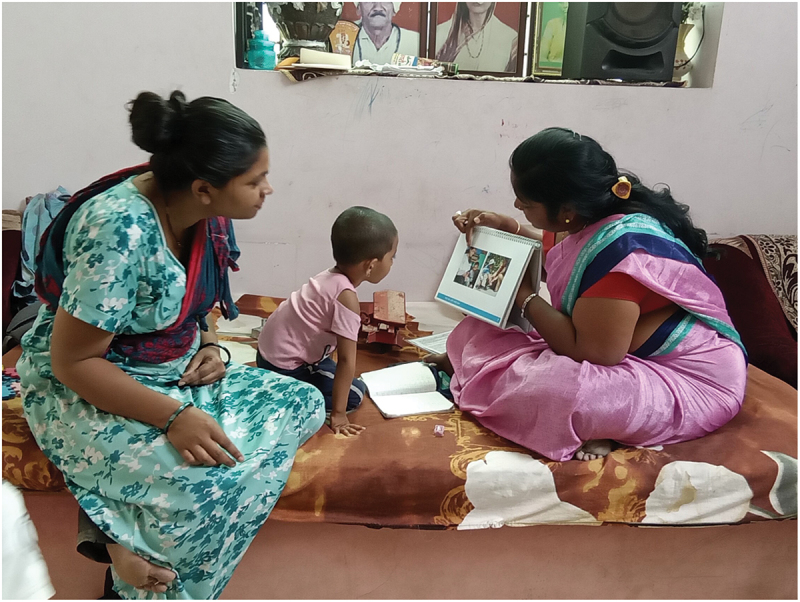
The GMCD Flipchart for individualized developmental recommendations and illustrative local photos in use by a rural frontline worker in India.

So as to retain the individualized developmental support, the adaptation team created a pictorial job aid called the GMCD Flipchart, where the recommendations for supporting a child’s development and accompanying locally tailored photographs can be shown directly to families. Recommendations are arranged to match the functioning level of the individual child across the GMCD’s seven developmental domains. The Flipchart guides frontline workers to the corresponding recommendations page based on the assessment and interpretation of the child’s functioning on the GMCD (Figure 3). Additional complementary job aids produced included tools for calculating chronological age in months and corrected age for prematurity based on birthweight. In team discussions consensus was that the impact on intervention fidelity from losing some automatic decision support workflows in the app would be offset by gains in acceptability and uptake by frontline workers from a tool they were more comfortable with.

### Module 3a: changes in packaging/materials (Modification of referral/triage processes)

In the original GMCD intervention for use with physicians and other professionals, frequency of monitoring and support visits with the GMCD was highly individualized and relied on clinician judgment. The adaptation teams decided that this approach should be revised since frontline workers have less training and experience in making clinical judgements. The modified approach helped frontline workers to classify children by predefined health and psychosocial risk factors and the presence or absence of developmental delays. Children without risk factors or developmental delays receive GMCD monitoring and support every three months, while those with risk factors or developmental delays receive monthly visits. Clear indications for referral to locally available resources are provided. The list of local developmental support resources including external services (such as physical therapy or speech therapy) and referral centers was compiled by each country team.

### Module 3a: training elements, pacing, structure

Refinements to the GMCD training strategy specifically for frontline workers were implemented. The most important of these strategies included:

- reductions in the amount of text on training slides, text replaced by visual representations and photos of families with increased emphasis on involvement of fathers and extended family
- contextualization of the concepts and skills being taught within the training curriculum
- multiple breaks and interactive games throughout each training day to maintain engagement and prevent fatigue
- expanded practice time for recognizing developmental milestones and for coding and interpretation of the GMCD results
- supplementary practice sessions for frontline workers with absences or those needing additional support.

### Module 3a: lengthening training (Development of post-training support strategy)

Post training support for physicians and other professionals in prior iterations has typically been individualized to context and skill level. A commonly used strategy has been remote review of self-recorded video of GMCD administration by a certified GMCD trainer. Given the rural nature of the settings here, as well as confidentiality and connectivity concerns, alternative methods were developed.

First, the adapted training methods gave training teams more direct observation time with trainees. For each training day, one hour was devoted to reflection on the prior session and review of homework practice and at the end of each day, one hour was given for practice with caregivers. This allowed for supportive feedback in real time. Subsequently, in India, post-training support involved monthly group meetings facilitated by trainers for 3 consecutive months along with a WhatsApp support group and monthly individual phone calls. In Guatemala, post training-support utilized a WhatsApp support group and face-to-face support visits to frontline workers in their place of work.

## Training of GMCD trainers as a part of co-creating adaptation

One important final element of new GMCD country implementations is development of a local cadre of trainers who then are able to train and certify other frontline workers. Key professional characteristics of GMCD trainers are given in Table 2.

**Table 2.** Desired personal and professional characteristics of GMCD trainers.

Domain	Characteristics
Personal	Compassionate Humble Open, eager to learn Patient Supportive Gives positive feedback Fosters confidence
Professional Experience	Service provider Experience working with young children and families Experience or interest in working with children with developmental difficulties Experience conducting reflective, experiential adult education
Leadership	Team building Communication Time management Planning and organization
GMCD Content	Received GMCD Service Provider and GMCD Trainer Certification Eagerness to apply strengths-based, family-centered approaches

In both countries, local trainers were trained early in the adaptation process. This meant they were then able to participate as active members of the co-creating team process described above. This process began with international master trainers conducting face-to-face-training of new trainers on GMCD package using unadapted materials.

In total, each trainer practiced at least 20 GMCD administrations achieving a reliability greater than 90% as assessed by master trainers on self-recorded administration videos.

Trainers were then also observed in person by master trainers as they delivered the pilot frontline worker training and given constructive feedback. Next, international master trainers provided online coaching and debriefing to each local team as they delivered the frontline worker pilot training described below. Experiences during these sessions were shared across the two country teams, leading to a single unified frontline worker package (Figure 1, Steps 7–8). In total, 3 trainers in India and 6 in Guatemala completed the trainer certification process. All trainers identified as female; the median age was 39.8 (range 28–48) years, and the median clinical experience was 11 (range 2–21) years. Their educational backgrounds included dentistry, medicine, nursing, psychology, and social work.

## Results from pilot training

### Frontline worker pilot training in India

Pilot training was conducted with 16 frontline workers (Anganwadi workers employed by ICDS). All trainees identified as female, with a median age of 47.5 (range 38–60) years. Training occurred in a rural public health training center and was delivered in-person one day per week for 6 consecutive weeks. Three GMCD trainers as well as 3 training assistants were present for all sessions. Training was in Marathi.

### Frontline worker pilot training process in Guatemala

Training was carried out with one rural non-governmental organization providing health and nutrition programs. Six frontline workers participated (5 female, 1 male), with a median age of 40.6 (range 35–50) years. Training again took place one day per week for 6 weeks. Six GMCD trainers participated, with two trainers leading each day’s activities. Training was in Spanish.

### Feedback and lessons learned from pilot training

Pilot training provided an opportunity to field test the adapted GMCD training package with frontline workers. Lessons learned from the pilot training were summarized by narrative review of daily written team notes, which included both staff observations and summaries of group-based end-of-day feedback. In addition, during the India pilot, frontline workers collaborated on the final day to produce a ‘radar plot’ of improvements in their knowledge and skills.

Key practical problems encountered and their solutions were as follows: (1) Content pacing needed improvement, and trainers moved some content from ‘heavy content’ days to lighter content days. (2) The pilot made frequent use of videos to teach essential GMCD skills. However, frontline workers had difficulty engaging with the large volume of video material, and some of this was replaced by role-play demonstrations by trainers. (3) It was common for spirited large-group discussion to cut into small group practice time. Therefore, large group discussion was kept focused and short.

**Figure 4.**
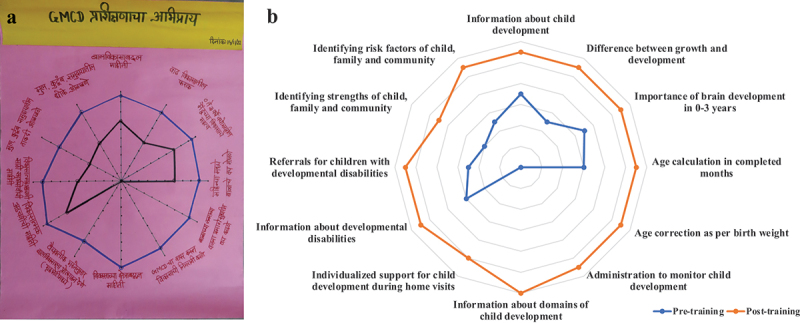
Example of collaborative group self-assessment using a “radar plot” activity in India. Inner line represents group’s consensus assessment of their skills before and outer line represents assessment of skills after completing GMCD training. (a) Original radar plot in Marathi (b) Translated to English.

In terms of qualitative participant feedback, in both countries sessions were enthusiastically received and resulted in positive self-assessments of improvements in knowledge. Figure 4 shows a collaboratively completed ‘radar plot’ from the last day of the India pilot showing large perceived pre-/post-improvements in most important knowledge domains.

## Discussion

In this Methods Paper, we describe in detail the adaptation of the International Guide for Monitoring Child Development (GMCD) for use in rural communities in India and Guatemala. We have developed an eight-step participatory co-creating approach to adapting interventions for task-shifting to frontline workers, and have used an implementation science framework for documenting this adaptation. We aim to provide a useful roadmap to inform future participatory intervention development or task-shifting efforts. Our approach is responsive to calls for participatory and consensus-building approaches to advance SDG goals [11].

Some of the adaptation decisions we document here are commonplace and described in other studies, such as language translations, tailoring of materials, and use of adult learning methods [19,20]. However, many others – such as formatting of job aids and post-training support plans – are less well described. The intensity of multidisciplinary collaboration, including direct and timely collaboration with frontline workers, allowed these insights to emerge early. Although participatory planning such as we describe is time consuming, it can prevent unanticipated problems from emerging after implementation has already begun. It can also render the intervention more acceptable and sustainable by building community ownership.

Our task-shifting implementation involves stakeholders from two very different country contexts, India and Guatemala [5]. In addition, the frontline workers in Guatemala are employed in the private sector by nongovernmental organizations, whereas the frontline workers in India are employed by the governmental ICDS program. This is by design, as our goal is to develop an adaptation that can work in different delivery contexts. Therefore, we believe it is likely that this GMCD adaptation will be sufficiently generalizable in terms of overall curriculum, training strategy, and support plans to be utilizable in most other country contexts. However, this assertion will require empirical validation as we and others scale up GMCD interventions in the coming years.

Finally, in this case study, we use an implementation science framework (FRAME-IS) to document our adaptation work [12]. Frameworks are commonly used to document adaptations in high-income settings, but their implementation in LMICs has been limited. Use of FRAME-IS in our case study will facilitate dissemination of our approach and lessons learned to other practitioners.

Our case report here has both strengths and limitations. The use of the FRAME-IS approach allowed us to comprehensively document our adaptations to the frontline workers in preparation for task-shifting of the GMCD intervention. The collaborative and iterative adaptation approach that incorporated perspectives from two countries increases the generalizability and utility of our lessons learned. A limitation of the current report is the small size of the training pilot, meaning that additional practical challenges may emerge when scaling up the implementation. Another limitation is the lack of prospective measures of acceptability, appropriateness, and feasibility before and after this adaptation process, meaning that we cannot make rigorous comparisons between our currently described GMCD adaptation for frontline workers with preexisting versions. An additional limitation is that our project has focused on adaptations for rural contexts, meaning that some material may not be optimal for children and families in urban settings.

## Conclusions

In this paper, we describe our co-creating, participatory approach to adapting the GMCD for task-shifting to frontline workers in India and Guatemala. Such co-creating approaches are an efficient way to ensure stakeholder and frontline worker engagement, identify barriers and develop solutions prior to full-scale implementation, and increase the generalizability of an adaptation to other contexts. Such approaches also help in building community ownership which is an essential step for community empowerment in turn. Lastly, use of the FRAME-IS approach can help to structure the documentation of adaptation efforts in LMICs.
